# Pulmonary Micro-Ecological Changes and Potential Microbial Markers in Lung Cancer Patients

**DOI:** 10.3389/fonc.2020.576855

**Published:** 2021-01-18

**Authors:** Zhuonan Ran, Jiexing Liu, Fen Wang, Caiyan Xin, Bin Xiong, Zhangyong Song

**Affiliations:** ^1^ The Second Department of Pulmonary and Critical Care Medicine, The Affiliated Hospital of Southwest Medical University, Luzhou, China; ^2^ School of Basic Medical Sciences, Southwest Medical University, Luzhou, China; ^3^ Molecular Biotechnology Platform, Public Center of Experimental Technology, Southwest Medical University, Luzhou, China

**Keywords:** lung neoplasms, genomics, biodiversity, biomarker, high-throughput sequencing

## Abstract

The relationship between the microbiome and disease has been investigated for many years. As a highly malignant tumor, biomarkers for lung cancer are diverse. However, precision of these biomarkers has not yet been achieved. It has been confirmed that lung microecology changes in lung cancer patients compared with healthy individuals. Furthermore, the abundance of some bacterial species shows obvious changes, suggesting their potential use as a microbial marker for the detection of lung cancer. In addition, recent studies have confirmed that inflammation, immune response, virulence factors, and metabolism may be potential mechanisms linking the microbiome with carcinogenesis. In this review, microbiome studies of lung cancer, potential mechanisms, potential microbial markers, and the influence of the microbiome on the diagnosis and treatment of lung cancer are summarized, providing theoretical strategies for the diagnosis and treatment of lung cancer.

## Highlights

- The relationship between the pulmonary microbiome and lung cancer has been recognized.
- The potential mechanisms linking the microbiome with carcinogenesis are summarized.
- Potential microbial markers of some bacterial species for lung cancer are highlighted.

## Introduction

In recent years, the relationship between the microbiome and disease has attracted wide attention. Many investigations have revealed that pathogen infection can lead to various diseases (1–3). For example, the correlation between *Helicobacter pylori* and gastrointestinal diseases has been confirmed (4). With the development of sequencing technology, the relationship between the pulmonary microbiome and health is being gradually recognized (3, 5, 6). Compared with healthy individuals, lung cancer patients exhibit changes in the abundance of different microbial species in the lung microbiome, which can cause an imbalance in the immune response (7, 8). Furthermore, inflammation caused by an imbalanced immune response can promote the development of various cancers, including ovarian cancer (9), colon cancer (10), and stomach cancer (11). It has been reported that approximately 15% of cancers are associated with chronic inflammation (12, 13). Chronic inflammation of the pulmonary system has also been reported to increase the risk of lung cancer (14, 15). However, until now, it has been challenging to reveal the mechanisms underlying alterations of the lung microbiome and to confirm their contributions to the development of lung cancer. In this review, the changes in the pulmonary microbiome in lung cancer are summarized. The relationship between the imbalanced immune response, caused by changes in pulmonary microecology, and lung cancer is also explored. In addition, potential microbial markers for lung cancer are highlighted, which will provide a new viewpoint for early screening and diagnosis of lung cancer.

## Lung Cancer

As a highly malignant tumor, the incidence and mortality of lung cancer is high (16, 17). The 5-year survival rate of patients with non-small cell lung cancer (NSCLC) is 21%, while the 5-year survival rate of patients with small cell lung cancer (SCLC) is only 7% (18). The predisposing factors and pathogenesis of lung cancer are complex and have not been completely defined. A recent study found that the presence of inflammation-inducing bacterial endotoxin lipopolysaccharide (LPS) in smoke can cause genetic changes. There were significant changes in the expression levels of 1,064 genes following LPS exposure in mice. Of these genes, 859 genes were related to tumorigenesis and metastasis. These results suggested that inflammation-inducing factors in cigarette smoke can cause early epigenetic changes in the lung, which, together with other factors (*e.g.*, environmental, genetic), can cause lung cancer (19). A retrospective meta-analysis showed that previous pulmonary diseases, such as pulmonary tuberculosis, chronic obstructive pulmonary disease (COPD), emphysema, chronic bronchitis, and pneumonia, increase the risk of lung cancer (14). The release of tumor necrosis factor (TNF) caused by *Mycobacterium tuberculosis* infection leads to pulmonary inflammation, and pulmonary fibrosis induced by *M*. *tuberculosis* results in synthesis of extracellular matrix (ECM) components. Thus, pulmonary inflammation and the ECM may be involved in the development of lung cancer (20). Epidemiological evidence suggests that tissue damage caused by inflammation can initiate or promote the development of lung cancer (21). These studies suggest that chronic inflammation is also a predisposing factor for lung cancer. Moreover, microorganisms have also been shown to influence the curative effect of lung cancer treatments. Programmed cell death-1 (PD-1)/programmed cell death 1 ligand 1(PD-L1) inhibitor antibodies can effectively improve the 5-year survival rate and overall survival of advanced NSCLC (22, 23). Further investigations showed that the efficacy of anti-PD-1 cancer treatment would be affected by intestinal microflora. In a germ-free mouse model, fecal microbiota transplantation ameliorated the antitumor effects of PD-1 blockade (24–27). Fecal bacteria transplantation can also enhance the curative effect of chemotherapy drugs (28, 29). In summary, current data illustrates that the microbiome is associated with the development of lung cancer.

## Microorganisms, Inflammation, and Cancer

### Microbiome

The microbial community of the human respiratory tract can be roughly divided into oral/nasal, upper respiratory, and pulmonary microbiomes. Due to direct communication with the outside, the oral and nasal cavity often changes with the environment, which in turn affects the microbial community of the upper respiratory tract. With the development of the endoscopic technique and gene sequencing technology, researchers have confirmed that healthy lungs are teeming with microbes, and lung microbes have been divided into the lung microbiome and lung mycobiome (30). Oral “microaspiration” has been shown to play an important role in the colonization of lung microorganisms. Comparison between oral specimens, protected specimen brushing (PSB), and bronchoalveolar lavage (BAL) specimens found that oral microflora and pulmonary microflora are similar, which confirmed that the colonized microflora in the lungs of healthy individuals migrated from the microaspiration of oral microflora (31–33). Sputum, BAL, and lung biopsy are commonly used to obtain specimens of lung microorganisms. Pollution of oral and upper respiratory tract can be avoided in BAL samples obtained by bronchoscopy and lung biopsy (31). However, these procedures are invasive, not readily available, and cause patient discomfort, and thus, their use has been limited in the study of the pulmonary microbiome. Although sputum may be contaminated by microflora from the oral and upper respiratory tract, the characteristics of the microbial community detected in sputum were closely related to tobacco smoking, severity of disease, pulmonary infection, and antibiotics use (34–36). Sputum is commonly used in many investigations because it is easily obtained, non-invasive, and reproducible (37, 38). The isolation and identification of lung microorganisms can be conducted by culture or non-culture methods. However, approximately 95% of lung microorganisms could not be detected by culture methods (39).

Investigations of the lung microbiome are currently unfolding. Analysis of sputum, BAL, oral, and nasopharyngeal specimens from healthy individuals found that there was no significant difference in different parts of the respiratory tract. Firmicutes and Bacteroides were found to be dominant in the lung microbiome of healthy individuals (40, 41). In terms of fungi, sequencing of oral washes (OWs), induced sputa (IS), and BAL from healthy individuals found that the mycobiomes of OWs, IS, and BAL had both common organisms as well as distinct members. Overlapping of BAL and OWs communities in healthy hosts suggested that lung microbes might originate from the mouth. *Candida* was the dominant species in OWs and IS (42). These studies show that even in the pulmonary systems of healthy individuals, the microbial community structure and composition is unique.

The microbiome not only acts on the colonized organs locally, but also on the whole body through inflammation, immunity, metabolism, and other mechanisms. For example, the increase in urea concentration in patients with chronic kidney disease will cause a change in intestinal flora and promote the production of intestinal endotoxin, which will adversely affect kidney function (43). Intestinal flora is involved in the pathogenesis of non-alcoholic fatty liver disease by affecting intestinal barrier function, choline metabolism, bile acid synthesis, and production of short-chain fatty acids and amino acids (44). The gut microbiota can increase the concentration of circulating short-chain fatty acids (SCFAs) by metabolizing the fiber. High circulating levels of SCFAs can protect the lung from allergic inflammation by inhibiting histone deacetylase and inducing myelopoiesis (45, 46). This indicates that the microbiome not only acts on the colonized organs locally, but also acts on the whole body through inflammation, immunity, metabolism, and other mechanisms.

### Microbiome and Cancer

A link between microbial infections and the development of cancer has been demonstrated. COPD is an important independent risk factor for lung cancer (47, 48). Chronic inflammation is a key characteristic of COPD (49). In most stable COPD patients also exists potentially pathogenic microorganism colonization (50, 51). Inflammation, oxidative stress, immunity, and DNA damage may increase the risk of lung cancer in COPD patients (52). This suggests that chronic respiratory infection may increase the body’s susceptibility to carcinogens and results in a higher risk of lung cancer. A meta-analysis indicated that infection with *M. tuberculosis* may increase the risk of lung cancer (14). *H. pylori* infection may also promote the occurrence and development of lung cancer (53), suggesting that pathogenic microbial infections can increase the risk of lung cancer. Microorganisms can promote the occurrence of lung cancer through inflammation, immune responses, virulence factors, and metabolism; inflammatory factors, such as interleukin (IL), tumor necrosis factor (TNF)-α, and cyclooxygenase (COX)-2, are closely related to carcinogenesis (12). IL-4 can promote tumor growth by inducing cathepsin protease activity in tumor-associated macrophages (54). In addition, NSCLC cells underwent a gradually progressing epithelial-to-mesenchymal (EMT) phenotype following exposure to IL-1β, an abundant proinflammatory cytokine in at-risk for lung cancer pulmonary and lung tumor microenvironments. EMT and EMT-associated phenotypes are related to cell invasion, PD-L1 upregulation, and chemoresistance (55). The inflammatory cytokine TNF-α can stabilize Snail through activation of the nuclear factor-kappa B (NF-*κ*B) pathway, which plays a critical role in inflammation-induced EMT and cancer cell migration, invasion, and metastasis (56). COX-2 stimulates the RhoA/Rho kinase pathway, which leads to disruption of tumor cell adherens junction formation and contributes to tumor progression (57). COX-2 is also overexpressed in NSCLC tissue and correlates with an unfavorable prognosis (58). In summary, pathogenic microorganisms infection can promote the release of inflammatory cytokines and aggravate local inflammatory response, thus promoting the occurrence and development of tumors.

The positive rate of the *H*. *pylori* (HP) in gastric cancer patients was positively correlated with the expression of macrophage migration inhibition factor (MIF) (59). The expression of *Mif* can promote cell transformation, tumor cell proliferation, and metastasis. Moreover, the expression of *Mif* was shown to be upregulated in NSCLC (60). The isolation and characterization of individual circulating tumor cells (CTCs)-associated white blood cells (WBCs) (CTCs–WBCs) as well as corresponding cancer cells within each CTCs–WBCs cluster showed that neutrophils and CTCs interact within the blood, and this interaction promotes cell cycle progression, which enhances the possibility of tumor metastasis of CTCs (61). COPD is at high risk of lung cancer (48). *Non-typeable Haemophilus influenzae* (NTHi) colonization can increase the frequency of exacerbations of COPD (62). Compared with the air-exposed group, when *IL-17C* gene-deficient metastatic lung cancer mice were exposed to NTHi, there was a significant reduction in the proliferation, growth, and number of tumor-associated neutrophils, suggesting that IL-17C plays a role in the development of neutrophil-mediated lung cancer (63).

*Escherichia coli* becomes highly pathogenic following the acquisition of virulence factors, including the protein toxin cytotoxic necrotizing factor 1 (CNF1), which induces the expression of *COX2*, activates the transcription factor NF-*κ*B, and promotes cellular motility, and thereby promotes tumor development (64). CNF1-induced bladder cancer cells secrete vascular endothelial growth factor (VEGF), leading to subsequent angiogenesis in the cancer microenvironment (65). Cytolethal distending toxin exposure leads to a unique cytotoxicity and induces a cell cycle arrest dependent on the DNA damage response (66). Moreover, enterotoxigenic *Bacteroides fragilis* and *B*. *fragilis* toxin gene was upregulated in patients with precancerous and cancerous lesions (67). Besides, microbial fermentation may have negative consequences owing to the generation of potentially toxic and cancer-promoting metabolites, such as ammonia, amines, phenols, sulphides, and nitrosamines (68). The microbiota modulates the enterohepatic circulation of estrogens through their ability to deconjugate estrogens, thus affecting circulating and excreted estrogen levels, and the risk for development of estrogen-dependent cancers (69). Overall, the role of inflammation and immunity in lung cancer development has been established; however, the correlation and interaction between pulmonary microorganisms and lung cancer are still not clear.

### New Directions for Cancer Treatment

There are new directions in cancer treatment with improved understanding of the correlation among microbes, inflammation, and cancer. For example, meroterpenoids, isolated from the brown seaweed *Cystoseira usneoides*, significantly reduced the production of TNF-α, IL-6, and IL-1β, suppressed *COX-2* expression, and displayed higher cytotoxic activities against lung cancer cells compared with normal lung cells (70). COX-2 can also enhance gefitinib resistance and NSCLC metastasis *via* PI3K-AKT silencing, which is a novel therapeutic strategy to overcome gefitinib resistance in NSCLC cells (58). On the other hand, a curative effect was observed following transplantation of the dominant intestinal flora of patients into the intestinal tract of a lung cancer mouse model (28). Smaller tumors and an improved survival rate were observed in lung cancer mice treated with cisplatin combined with *Lactobacillus* bacteria (29). Moreover, antibodies targeting cytotoxic T-lymphocyte antigen (CTLA)-4 have been successfully used as cancer immunotherapy; the anticancer activity of CTLA-4 depends on intestinal flora. Investigations have confirmed that *Bifidobacterium* enhances the therapeutic effects of PD-L1 and CTLA-4 by altering the activity of dendritic cells and enhancing CD8^+^ T cell activation (71, 72). A recent investigation found that there are many intestinal fungi in the tumor tissues of pancreatic ductal adenocarcinoma (PDA) cells. Furthermore, the elimination of fungi can inhibit the growth of tumors in PDA mice and improve the effects of chemotherapy (73). The antitumor effects of the microbiome may be influenced by the type and strength of the immune response and cross-reactions between the microbiome and tumor antigen (74). However, further exploration of the use of microorganisms in the treatment of tumors is necessary.

## Lung Cancer Biomarkers

### Development of Lung Cancer Biomarkers

There are many biomarkers for lung cancer. For example, the sensitivity of a panel of autoantibodies to tumor-associated antigens was higher than a single antigen. However, only 40% of primary lung cancers can be identified by testing with a panel of autoantibodies in peripheral blood (75). Comprehensive assessment of suspected lung cancer using circulating microRNAs (miRNAs) can effectively reduce the false positive rate of LDCT and can also be used to monitor cancer recurrence (76). The detection of mature mir-21 in the sputum of 23 lung cancer patients showed that the expression of mir-21 was significantly higher than that of non-cancer patients, with a detection rate of 69.66% and specificity of 100.00% (77). Circulating tumor DNA is frequently present in patients with advanced NSCLC, which can be used to assess the clinical benefits of Nivolumab (78). However, it can only be used to assess recurrence after primary surgery for NSCLC (79). Elevated levels of IL-6, IL-8, and C-reactive protein are associated with the diagnosis of lung cancer. The levels of IL-8 begin to increase 5 years prior to the diagnosis of lung cancer. Therefore, the combination of IL-6 and IL-8 could be useful for evaluating the prognosis of lung cancer (80). Detection of exhaled breath volatile organic compounds by special instruments may also be useful in the diagnosis and classification of cancer (81). Compared with cancer-free specimens, measurement of metabolites in sputum samples of lung cancer patients revealed that levels of isobutyl decanoate, putrescine, diethyl glutarate, and cysteamine were significantly changed (82), suggesting that metabolites in sputum may act as biomarkers for lung cancer. Although detection biomarkers for lung cancer are diverse, precision by these biomarkers has not yet been achieved. Further research should be performed in large and well designed trials as most data is retrospective or in small series.

### Microbiome and Potential Microbial Markers of Lung Cancer

At present, several investigations have confirmed changes in lung microecology in lung cancer; however, different changes in the bacterial flora of lung cancer patients were observed in different specimens. BAL samples from lung cancer patients had elevated levels of Firmicutes and TM7 kingdom; levels of the genera *Veillonella* and *Megasphaera* were also increased significantly (7). PSB samples from lung cancer patients revealed that the levels of *Streptococcus* in lung cancer were significantly higher compared with the non-cancerous control. An upward trend of *Neisseria* in cancerous lesions and *Staphylococcus* and *Dialister* displayed a decreasing trend in the non-cancerous group compared with the cancer group. Significantly decreased microbial diversity in lung cancer has also been observed (8). Moreover, the abundance of Firmicutes (*Streptococcus*) and Bacteroides (*Prevotella*) was significantly increased in the lung tissues of patients with surgically treated lung cancer compared with emphysema patients (83). 16S rDNA sequencing analysis showed that the abundance of salivary species in patients with lung cancer differed from the non-cancerous group. Compared with the non-cancerous group at the genus level, the abundances of *Capnocytophaga*, *Selenomonas*, and *Veillonella* were higher and the abundance of *Neisseria* was lower in lung cancer patients. At the species level, compared with the squamous cell carcinoma group, the abundances of *Streptococcus* and *Porphyromonas* in adenocarcinoma patients were higher. *Neisseria*, *Capnocytophaga*, and *Veillonella* may be potential biomarkers of lung cancer (84). Another metagenomic sequence analysis found that the abundance of *Granulicatella adiacens*, *Streptococcus intermedius*, *S. viridans*, and *M. tuberculosis* in the sputum of patients with lung cancer was significantly higher compared with the non-cancerous group. The dominant trend of *G. adiacens* is related to *Enterococcus* sp.130, *E. coli*, *S. intermedius*, *Acinetobacter junii*, *S. viridans*, and *S.* sp.6. Significant changes in level 2 and 3 functions, such as urea cycle, putrescine utilization, and intracellular resistance, were also observed from negative to positive lung cancer. However, compared with the non-cancerous group, there was no significant change in sputum species diversity in lung cancer patients (85). Compared with the non-cancerous group, the analysis of 16S rDNA high-throughput sequencing of sputum from non-smoking female lung cancer patients found that the relative abundances of *Granulicatella* (6.1%), *Abiotrophia* (1.5%) and *Streptococcus* (40.1%) were significantly increased (86). Compared with squamous cell carcinoma, the abundances of *Thermus* and *Ralstonia* were higher and lower, respectively, in adenocarcinoma. Alpha diversity is statistically significantly higher in patients with adenocarcinoma (87). In addition, *TP53* mutations have been shown to be related to the abundance of microorganisms in squamous cell carcinoma (88). Further investigation found that a number of microorganisms, including *Acidovorax*, *Klebsiella*, *Rhodoferax*, *Comamonas*, and *Polarmonas*, were more abundant in squamous cell carcinoma with TP53 mutations (89).

**Table 1** shows a summary of the relevant studies; overall, the pulmonary microbiome in lung cancer patients differs from that in tumor-free patients. In terms of biodiversity, the results are inconsistent, which may be related to small sample sizes and clinical sampling differences. Several studies have shown that the abundance of Streptococcus (Firmicutes) in lung cancer patient samples was significantly increased. *In vitro*, investigations have revealed that *S. pneumoniae* can cause damage to alveolar epithelial cells (90). Similar results have confirmed that *S. pneumoniae* directly inhibits purinergic signaling by inducing purinergic receptor P2Y2 phosphorylation and internalization, which can lead to suppression of the calcium response of alveolar epithelial cells to ATP and affect the cellular integrity and function (91). Streptococcus may promote the development of lung cancer, although further experiments are needed for verification.

**Table 1:** Microorganisms with varying abundance in lung cancer patients.

Sample type	Control group	Main finding	Reference
BAL	Tumor free	**Phylum**: Firmicutes and TM7 increased in lung cancer group; **Genus**: *Veillonella, Megasphaera, Atopobium*, and *Selenomonas* increased in lung cancer group; Alpha diversity increased in lung cancer group;	Lee et al. (7)
PBS	Healthy	**Genus**: *Streptococcus, Haemophilus*, and *Neisseria* increased in lung cancer group; *Staphylococcus* and *Dialister* decreased in lung cancer group; Alpha diversity decreased in lung cancer group;	Liu et al. (8)
Lung tissue	Emphysema	**Phylum**: Firmicutes, Bacteroidetes, and Actinobacteria increased in lung cancer group; Proteobacteria decreased in lung cancer group; **Genus**: *Streptococcus, Prevotella*, and *Bifidobacterium* increased in lung cancer group; *Acinetobacter, Acidovorax*, and *Diaphorobacter* decreased in lung cancer group; Alpha diversity increased in lung cancer group;	Liu et al. (83)
Salivary	Tumor free	**Order**: Flavobacteriales and Burkholderiales increased in lung cancer group; Bacteroidales decreased in lung cancer group; **Family**: Veillonellaceae increased in lung cancer group; Lachnospiraceae decreased in lung cancer group; **Genus**: *Capnocytophaga, Selenomonas*, and *Veillonella* increased in lung cancer group; *Neisseria* and *Streptococcus* decreased in lung cancer group; The abundances of *Streptococcus* and *Porphyromonas* in adenocarcinoma patients was higher than in squamous cell carcinoma patients;	Yan et al. (84)
Sputum	Tumor free	**Species**: *Streptococcus viridans, Streptococcus intermedius, Granulicatella adiacens, Mycobacterium tuberculosis*, and *Mycobacterium bovis* increased in lung cancer group; Alpha diversity was not significantly different in the lung cancer group;	Cameron et al. (85)
Sputum	Tumor free	**Genus**: *Streptococcus, Abiotrophia*, and *Granulicatella* increased in lung cancer group; *Leptotrichia, Sphingomonas* decreased in lung cancer group;	Hosgood et al. (86)
Lung tissue	_	Adenocarcinoma *vs.* squamous cell carcinoma: abundance of *Thermus* was higher in adenocarcinoma, abundance of *Ralstonia* was lower in adenocarcinoma; Alpha diversity was statistically significantly higher in patients with adenocarcinoma;	Yu et al. (87)

BAL, bronchoalveolar lavage; PSB, protected specimen brushing; “-”, null.

## Conclusions and Prospects

Over the past few decades, significant progress has been made in our understanding of the relationship between the microbiome and lung cancer. From the above-mentioned investigations, we know that the microbiota of lung cancer patients is changed, and the abundance of certain bacteria in lung cancer patients is significantly increased. However, how the changes in the composition and metabolism of the microbiome contribute to lung cancer development remains largely unexplored. As the small sample sizes, the investigations of the microbiome in lung cancer remain at analysis of the rule of microbiome change. Further investigations are needed to clarify the mechanism underlying the influence of the microbiome on the occurrence and development of lung cancer and interactions between the microbiome and lung cancer. If the increased abundance of microorganisms in lung cancer patients promotes the progression of lung cancer, then early monitoring and intervention for the screening and treatment of lung cancer patients become particularly important. Further understanding of these mechanisms will shed light on early screening, diagnosis, and treatment of lung cancer.

## Author Contributions

ZNR, CYX, and ZYS wrote the manuscript. JXL and FW critically revised it. ZYS and BX supervised the work. All authors contributed to the article and approved the submitted version.

## Funding

This research was funded by the National Natural Science Foundation of the China (31701127), Science and Technology Project of Sichuan (2019YJ0407, 2017JY0165), and Foundation of Affiliated Hospital of Southwest Medical University (2015-YJ011).

## Conflict of Interest

The authors declare that the research was conducted in the absence of any commercial or financial relationships that could be construed as a potential conflict of interest.

The reviewer [QY] declared a shared affiliation with the authors, to the handling editor at the time of review.
